# Phenomics approaches to understand genetic networks and gene function in yeast

**DOI:** 10.1042/BST20210285

**Published:** 2022-03-14

**Authors:** Clarence Hue Lok Yeung, Nil Sahin, Brenda Andrews

**Affiliations:** The Donnelly Centre and the Department of Molecular Genetics, University of Toronto, Toronto, Canada

**Keywords:** biological networks, genomics, high-throughput screening

## Abstract

Over the past decade, major efforts have been made to systematically survey the characteristics or phenotypes associated with genetic variation in a variety of model systems. These so-called phenomics projects involve the measurement of ‘phenomes’, or the set of phenotypic information that describes an organism or cell, in various genetic contexts or states, and in response to external factors, such as environmental signals. Our understanding of the phenome of an organism depends on the availability of reagents that enable systematic evaluation of the spectrum of possible phenotypic variation and the types of measurements that can be taken. Here, we highlight phenomics studies that use the budding yeast, a pioneer model organism for functional genomics research. We focus on genetic perturbation screens designed to explore genetic interactions, using a variety of phenotypic read-outs, from cell growth to subcellular morphology.

## Introduction

A principal goal of many phenomics projects is to shed light on the complex relationship between an organism's genotype and phenotype, thus enabling functional annotation of the genome (reviewed in [1–4]). The general approach involves assessing the consequences of gene perturbation, by mutation or chemical treatment, using a phenotypic read-out of choice. In this mini-review, we focus on the budding yeast, *Saccharomyces cerevisiae*, which has been a pioneering system for functional genomics studies, largely due to the availability of genome-wide tools for systematic analyses in isogenic backgrounds, and a highly engaged research community dedicated to making reagents and information freely available through the *Saccharomyces* Genome Database (SGD: https://www.yeastgenome.org, [5]). One key reagent set was constructed by a consortium of yeast labs more than two decades ago. The yeast deletion project involved the systematic replacement of each predicted open reading frame (ORF) in the genome with a selectable antibiotic resistance marker. This remarkable effort revealed that individual deletion of ∼5000 genes was compatible with haploid strain viability, while also defining a set of ∼1000 essential genes [6,7]. Several strain collections for studying the phenotypes associated with essential gene perturbation have also been constructed; the most widely used collections consist of strains expressing temperature-sensitive alleles that are hypomorphic at permissive temperatures and non-functional at higher temperatures [8–11].

As noted above, a key insight from the yeast deletion project is that most single-gene deletions are compatible with cell viability, a feature that seems to be broadly conserved [12]. This observation revealed the apparent genetic redundancy or buffering inherent to eukaryotic genomes and has catalyzed functional genomics projects aimed at exploring how genes interact to produce complex phenotypes. A genetic interaction occurs when the combination of two genetic mutations gives rise to an unexpected phenotype that deviates from the expected cumulative effects of both individual mutations (reviewed in [3]). Again, efforts to systematically explore genetic interactions have been pioneered using the budding yeast system. Below, we discuss phenomics approaches that have been used to map genetic networks in yeast.

## Fitness-based phenomics for mapping genetic networks

The most widely used measurement for scoring phenotypes in genome-wide perturbation screens is cell proliferation, due to the ease and scalability of cell growth assays. In budding yeast, arrayed mutant strains collections can be cultured on agar plates as colonies, and replica-pinned to various media. Colony size is then used as a quantitative assay of cell fitness, and a mutant phenotype can be immediately linked to the underlying genotype by its known position on the array [13,14]. The relative fitness of mutant strains can also be assayed after growth of pooled strains in liquid collection, since the mutant alleles in the yeast deletion collection feature a unique molecular barcode, enabling identification of the relative abundance of mutant strains using a barcode sequencing read-out [6,15].

**Figure 1.**
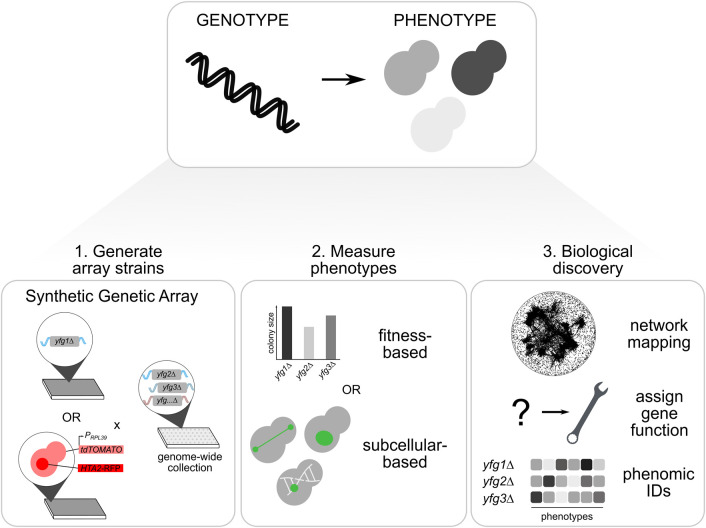
Illustration of the experimental pipeline for discovery and analysis of genetic interactions in budding yeast. A principal goal of phenomics studies is to understand the relationship between a cell or organism's genotype and phenotype. In budding yeast, arrays of single and double mutants, carrying gene mutations or fluorescent markers of interest integrated into the genome either through C-terminal tagging (e.g. *HTA2-RFP*) or a fluorescent reporter driven by a gene-specific promoter (e.g. *P_RPL39_-tdTOMATO)*, are constructed using an automated approach known as the Synthetic Genetic Array (SGA) method (panel 1). Following strain array construction, phenotypes are measured using a fitness-based (e.g. colony size) or subcellular-based (e.g. fluorescent reporter) read-out (panel 2). Finally, computational analyses enable the construction of genetic networks, assignment of novel gene function and the generation of multiparametric measurements, or phenomic IDs, specific to each genetic perturbation (panel 3). *yfg* = ‘your favorite gene’.

Over the past ∼two decades, genetic interactions in yeast have been systematically mapped using colony size as a phenotypic read-out [11,16–19]. Large-scale mapping of genetic networks was made possible by the development of an automated form of yeast genetics, termed Synthetic Genetic Array (SGA) analysis, that enables rapid construction of double mutant arrays (Figure 1) [16]. Colony size of single and double mutants is compared and deviations from the growth phenotype expected based on the single mutant phenotypes is calculated [13,20]. For large-scale and complex genetic interaction screens, automated image processing of mutant arrays is used to quantify colony size phenotypes and to control for batch effects [13,21]. Using this approach, a genome-wide genetic interaction network was mapped by examining all possible ∼18 million gene pairs for double mutants that were either less or more fit than expected [3,11]. The resultant genetic network consists of ∼550 000 negative genetic (synthetic lethal or sick) and ∼350 000 positive genetic (synthetic suppressive) interactions. To put these numbers in perspective, while there are ∼1000 essential yeast genes, in which a single mutation causes a severe growth defect, there are 500-fold more digenic combinations that lead to an extreme fitness defect. This ratio of negative digenic interactions to essential genes unequivocally illustrates the prevalence of genetic buffering [22] and the vast potential for combinatorial genetics to drive both cellular and organismal phenotypes.

**Figure 2.**
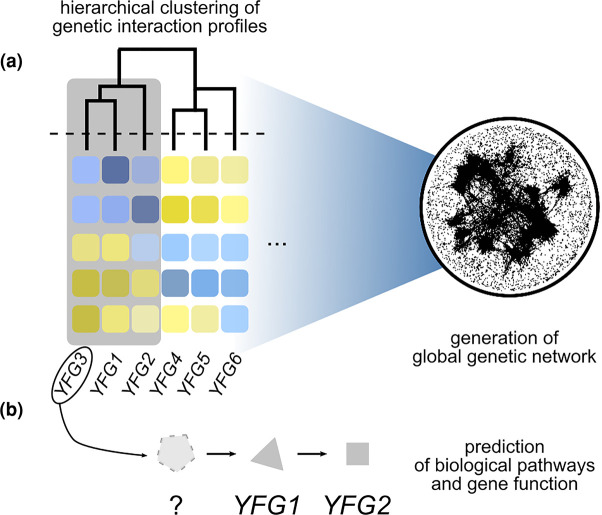
Hierarchical clustering of genetic interaction profiles enables the assembly of a global genetic interaction network. (**a**) Genes with similar genetic interaction profiles, based on fitness-based or single cell phenotypes, can be computationally clustered to identify groups of genes likely to share functions in common. A global network view can be produced by plotting genes on the network based on quantitative analysis of the similarity in their genetic interaction profiles. Genes that share a lot of genetic interactions in common are spatially closer together on the global network, whereas genes with less similar genetic interaction profiles are placed further apart. The resultant network can be functionally annotated to discover the functional information associated with the various clusters or groups on the network. (**b**) The position and connectivity of genes on the global genetic interaction network enables prediction of their participation in biology pathways or protein complexes, and of unknown gene function. For example, *YFG3* is predicted to participate in the same pathway as *YFG1* and *YFG2* by virtue of its similarity of genetic interaction profile. (*YFG* = your favorite gene).

The genetic interaction profile or set of genetic interactions associated with a particular gene perturbation can be clustered with other genetic interaction profiles to reveal a hierarchy of cell function (Figure 2) [11]. Genes that have similar genetic interactions profiles tend to be involved in the same bioprocesses, thus the position and connectivity on the global genetic network can be used to predict gene and pathway function. This property of quantitative genetic interaction profiles means that fitness-based profiles generated by other types of perturbations can also be mapped onto the genetic network, to reveal functional information. For example, a chemical-genetic profile, which consists of quantitative measurements of the sensitivity or resistance of yeast mutants to a specific compound, may resemble the genetic interaction profile associated with perturbation of its target [23,24]. A web-accessible database and visualization tool has been designed to facilitate data access and navigation of the global yeast genetic interaction network [25]. In addition to cell fitness, other phenotypes that rely on population-based phenotypes have been used to study genetic interactions in yeast, including transcriptional profiling to explore genetic interactions involving transcription factors, kinases, and phosphatases [26,27].

As noted above, the inclusion of barcodes marking mutant alleles in the yeast deletion collection enables the measurement of growth defects of pooled strain collections in competitive growth assays. Methods to map yeast genetic interactions in pooled cultures using barcode microarray or sequencing-based read-outs include dSLAM [28], GI mapping (GIM; [29,30]) and DNA barcode fusion (BFG-GI; [31,32]). Pooled approaches have been adapted to study the effects of GIs on the quiescence phenotype in response to different nutrient deprivations [33]. Barcodes are now commonly included in the design of libraries for systematic gene perturbation in other systems, including genome-scale guide RNA (gRNA) libraries for CRISPR–Cas9-mediated disruption of genes in human cells (reviewed in [34]). A flow cytometry-based read-out has also been used to map GIs following competitive growth of hypomorphic and wild-type alleles of essential yeast genes tagged with different fluorescent proteins by monitoring relative strain abundance after serial dilutions [8]. Recently, methods were developed to rapidly construct double mutants of yeast CRISPR interference (CRISPR-i) alleles, in which genes are repressed by dCas9-based recruitment of a repressor to their promoters, compatible with sequencing-based pooled growth assays [35]. In general, competitive growth assays can provide a more sensitive read-out of genetic interactions, complementing large-scale efforts to map GIs using colony size.

In addition to double mutant genetic interactions, fitness-based phenomics is being used to systematically explore more complex genetic scenarios. For example, the SGA method was recently adapted to survey triple mutant genetic interactions, providing insight into the prevalence of trigenic interactions, the mechanisms of retention of duplicated genes, and the functional relationships between distant biological processes [36–40]. In addition, measurements of GIs in a variety of stress and other environmental conditions revealed that the GI network is remarkably robust, with most conditional GIs being detectable in the reference network but exacerbated or suppressed in different growth conditions [41]. Finally, higher-order genetic interactions can be explored using XGA (‘X-gene’ genetic analysis), which involves ‘en-masse’ mating of a barcoded pool of wild-type cells with a multi-mutant strain deleted for genes of interest, followed by barcode sequencing of the progeny to discover genetic interactions [42]. Combinations of 16 yeast ABC transporter gene deletions revealed novel high-order genetic and chemical-genetic interactions, not evident from analysis of single and double gene knockouts [42].

## Cell biological phenomics to explore genetic networks and gene function

While fitness-based assays have enabled genome-wide mapping of genetic interactions, cell growth is not a precise phenotype, and population-based measurements preclude an assessment of population heterogeneity. Furthermore, most single deletions of non-essential genes and double mutant combinations do not cause a significant growth defect [11]. Specifically, ∼80% of deletion mutants grow comparably to wild-type (less than a 5% decrease in fitness relative to wild-type), and deletion mutants show a negative genetic interaction with ∼2% of other deletion mutants [11]. The potential of multi-parametric phenotypic measurements in yeast mutants to add to our view of gene and pathway function has been clear for more than 15 years. For example, the Ohya lab explored the yeast deletion collection for defects in the actin cytoskeleton, cell membrane and nucleus by immuno-staining of the relevant compartments, followed by image analysis [43]. The project revealed that ∼50% of yeast deletion mutants had a morphological phenotype, and comparison of phenotypic profiles allowed the assignment of genes to specific biological pathways [43]. This approach also enabled the identification of drug targets by obtaining and comparing chemically induced phenotypic profiles to mutant cell phenotypes. Importantly, the project led to the development of CalMorph, one of the first high-throughput image-processing software packages for the extraction of morphological features specifically from yeast cell images [44].

The roster of phenotypes amenable to genome-scale image analysis has expanded tremendously due to advances in high-throughput microscopy and computational image analysis, often referred to as high-content screening (reviewed in [45]). In budding yeast, the combination of high-content screening and automated yeast genetics has proven particularly powerful for exploring the genetic determinants of sub-cellular morphology. In addition to facilitating rapid construction of double mutant yeast arrays (see above), the SGA method can also be used to introduce fluorescent markers of any subcellular compartment of interest into arrayed collections of yeast mutants (Figure 1). Additional markers to provide spatial context, such as nuclear and cytoplasmic markers, can also be simultaneously introduced enabling cell segmentation and automated image analysis [46].

This general approach has been applied over the last decade or so, using various imaging systems and image analysis protocols, to discover phenotypes caused by single-gene perturbations for a spectrum of subcellular structures and processes including: (1) morphology of the mitotic spindle [47]; (2) determinants of nuclear shape [48,49]; (3) nucleolar size regulation [50]; (4) regulation of trafficking at endosomes [51]; (5) formation of DNA damage foci [52] and; (6) endocytosis compartment morphology and regulators of endocytosis [53–55]. The integration of innovative tools such as microfluidics into automated imaging pipelines has enabled temporal single-cell analysis in a high-throughput manner. These systems allow researchers to track the morphological changes that yeast cells go through during their entire replicative lifespan including aging and to investigate genetic and environmental factors that regulate lifespan [56,57].

Systematic morphological profiling of yeast mutants can reveal not only the roster of genes that influence the structure and function of subcellular organelles and compartments, but also the prevalence of phenotypic heterogeneity and pleiotropy. For example, a systematic analysis of four markers of the endocytic pathway using single cell imaging and automated image analysis revealed that many mutants were associated with multiple phenotypes, indicating that morphological pleiotropy is common for endocytosis compartments [53]. Incomplete penetrance was also prevalent, and single cell image analysis enabled exploration of underlying mechanisms, such as replicative age, organelle inheritance and stress response.

Comparable image analysis methods have been developed to exploit another key strain collection, the ORF-GFP array, for assaying proteome dynamics as a read-out for genetic or other perturbations. The GFP collections contains ∼4100 strains expressing different proteins tagged C-terminally with GFP, at their endogenous loci [58]. The GFP array has been extensively validated by the yeast community [59–62] and other collections of strains expressing RFP-tagged proteins, N-terminally tagged derivatives or tandem tags are also available [63–67]. These collections are unique resources, enabling assessment of the localization and abundance of most of the proteome in living cells using fluorescence microscopy. Computational image analysis tools have been developed that enable quantitative assignment of yeast proteins into 22 subcellular compartments and the construction of maps or ‘flux networks’ describing proteome changes in response to genetic [60] or environmental perturbations [40,68,69]. For example, automated image analysis of the ORF-GFP collection was recently used to study proteome plasticity in response to heat stress, revealing protein localization changes that may protect proteins from thermal instability and enable new functions [40]. Single cell image analysis promises to enable researchers to assemble ‘phenomic IDs’, or cellular proteomic signatures, based on changes in protein abundance and localization, in response to specific environmental insults, gene mutation, genetic interactions and cell states, such as cell cycle position.

## Conclusion and outlook

So far, except for colony size phenotypes, most of the phenomics assays that we've discussed have been used to survey single mutant phenotypes. However, there are several reasons to anticipate that more sensitive cell biological read-outs will provide new insights into genetic interactions and networks, beyond what has been learned from population-based measurements of cell growth:

1. First, some genes (∼20% of tested genes) associated with weak genetic interaction profiles are not represented on the global network or localize to peripheral, unstructured regions of the network, which are not enriched for genes associated with any specific function [11]. These genes may be refractory to genetic interaction profiling if a single gene perturbation or a more complex genetic interaction does not produce an obvious growth phenotype, demanding a more sensitive read-out to detect single and double mutant phenotypes.
2. Second, genetic interactions that produce an unexpected growth phenotype are rare (∼2% of gene pairs involving non-essential genes will exhibit a genetic interaction [41]), but functionally informative genetic interactions may be detectable by scoring more specific phenotypes. For example, a study of genetic interactions using a fluorescent marker of the DNA damage focus expanded the roster of mutants that show a genetic interaction with *SGS1*, a RecQ helicase, by ∼50%, relative to screens using a colony-size read-out [52]. Single-cell analysis of images of single and double mutants increases the resolution of cell phenotypes caused by GIs and may provide a mechanistic understanding of GIs that cannot be captured using colony size or fitness measurements.
3. Third, population-level measurements do not allow an analysis of cell-to-cell variability, which is a key consideration for predicting the consequences of genetic perturbation and genetic interactions [53,70].

Available yeast reagents and pipelines for automated image analysis can be used to map genetic networks by examining all proteins (the GFP collection) or specific markers of interest in double mutant (or multi-mutant) backgrounds. The complexities of biological networks demand continued development and application of new systems-level approaches, which can only be achieved in highly accessible model systems. Foundational work using the yeast system will continue to provide a clear conceptual and technical road map for mapping and interpreting quantitative phenotypes in other systems, including human cells, bringing us closer to unraveling the genotype-to-phenotype relationship.

## Perspectives

- Systematic phenotypic analysis, or phenomics, has been used to map global networks of genetic interactions and to quantitatively describe the consequences of gene perturbation in budding yeast. The prevalence of genetic buffering underscores the importance of studying genetic interactions and collecting multi-parametric measurements to further our understanding of the genotype–phenotype relationship in eukaryotic organisms.
- Based on population-level analyses of growth phenotypes associated with single and double-mutant genetic perturbations, a global genetic interaction network has been produced for budding yeast. The network defines the general principles of genetic interactions, providing a paradigm for mapping functional connections among genes and pathways. In parallel, analyses of cell biological phenotypes in single cells using automated imaging have revealed the potential for complex phenotypic read-outs to expand our view of gene function and the global genetic interaction network.
- Advances in imaging technology and computational resources will increase the throughput and complexity of phenomics projects, extending population-level studies to single-cell analyses and digenic interactions to multi-gene interactions. These efforts promise to provide a complete view of the genetic determinants of subcellular biology, and the prevalence and mechanisms of incomplete penetrance and cell-to-cell heterogeneity. Approaches developed in the budding yeast system will provide a proof-of-principle for both a technical and conceptual platform that should be readily adapted for parallel screening efforts to measure genetic interactions in mammalian cells using CRISPR–Cas-based genome editing technologies.

## Abbreviations

ORF: open reading frame
SGA: synthetic genetic array

## References

[1] Houle, D., Govindaraju, D.R. and Omholt, S. (2010) Phenomics: the next challenge. Nat. Rev. Genet. 11, 855–866 10.1038/nrg2897 21085204

[2] Lehner, B. (2013) Genotype to phenotype: lessons from model organisms for human genetics. Nat. Rev. Genet. 14, 168–178 10.1038/nrg3404 23358379

[3] Costanzo, M., Kuzmin, E., van Leeuwen, J., Mair, B., Moffat, J., Boone, C. et al. (2019) Global genetic networks and the genotype-to-phenotype relationship. Cell 177, 85–100 10.1016/j.cell.2019.01.033 30901552 PMC6817365

[4] Ohya, Y., Kimori, Y., Okada, H. and Ohnuki, S. (2015) Single-cell phenomics in budding yeast. Mol. Biol. Cell 26, 3920–3925 10.1091/mbc.E15-07-0466 26543200 PMC4710224

[5] Cherry, J.M. (2015) The saccharomyces genome database: a tool for discovery. Cold Spring Harb. Protoc. 2015, pdb top083840 10.1101/pdb.top083840 26631132 PMC5673599

[6] Winzeler, E.A., Shoemaker, D.D., Astromoff, A., Liang, H., Anderson, K., Andre, B. et al. (1999) Functional characterization of the *S. cerevisiae* genome by gene deletion and parallel analysis. Science 285, 901–906 10.1126/science.285.5429.901 10436161

[7] Giaever, G. and Nislow, C. (2014) The yeast deletion collection: a decade of functional genomics. Genetics 197, 451–465 10.1534/genetics.114.161620 24939991 PMC4063906

[8] Breslow, D.K., Cameron, D.M., Collins, S.R., Schuldiner, M., Stewart-Ornstein, J., Newman, H.W. et al. (2008) A comprehensive strategy enabling high-resolution functional analysis of the yeast genome. Nat. Methods 5, 711–718 10.1038/nmeth.1234 18622397 PMC2756093

[9] Ben-Aroya, S., Coombes, C., Kwok, T., O'Donnell, K.A., Boeke, J.D. and Hieter, P. (2008) Toward a comprehensive temperature-sensitive mutant repository of the essential genes of *Saccharomyces cerevisiae*. Mol. Cell 30, 248–258 10.1016/j.molcel.2008.02.021 18439903 PMC4130347

[10] Li, Z., Vizeacoumar, F.J., Bahr, S., Li, J., Warringer, J., Vizeacoumar, F.S. et al. (2011) Systematic exploration of essential yeast gene function with temperature-sensitive mutants. Nat. Biotechnol. 29, 361–367 10.1038/nbt.1832 21441928 PMC3286520

[11] Costanzo, M., VanderSluis, B., Koch, E.N., Baryshnikova, A., Pons, C., Tan, G. et al. (2016) A global genetic interaction network maps a wiring diagram of cellular function. Science 353, aaf1420 10.1126/science.aaf1420 27708008 PMC5661885

[12] Boone, C. and Andrews, B.J. (2015) Human genome. The indispensable genome. Science 350, 1028–1029 10.1126/science.aad7925 26612934

[13] Baryshnikova, A., Costanzo, M., Kim, Y., Ding, H., Koh, J., Toufighi, K. et al. (2010) Quantitative analysis of fitness and genetic interactions in yeast on a genome scale. Nat. Methods 7, 1017–1024 10.1038/nmeth.1534 21076421 PMC3117325

[14] Memarian, N., Jessulat, M., Alirezaie, J., Mir-Rashed, N., Xu, J., Zareie, M. et al. (2007) Colony size measurement of the yeast gene deletion strains for functional genomics. BMC Bioinformatics 8, 117 10.1186/1471-2105-8-117 17408490 PMC1854909

[15] Giaever, G., Chu, A.M., Ni, L., Connelly, C., Riles, L., Veronneau, S. et al. (2002) Functional profiling of the *Saccharomyces cerevisiae* genome. Nature 418, 387–391 10.1038/nature00935 12140549

[16] Tong, A.H., Evangelista, M., Parsons, A.B., Xu, H., Bader, G.D., Page, N. et al. (2001) Systematic genetic analysis with ordered arrays of yeast deletion mutants. Science 294, 2364–2368 10.1126/science.1065810 11743205

[17] Tong, A.H., Lesage, G., Bader, G.D., Ding, H., Xu, H., Xin, X. et al. (2004) Global mapping of the yeast genetic interaction network. Science 303, 808–813 10.1126/science.1091317 14764870

[18] Schuldiner, M., Collins, S.R., Thompson, N.J., Denic, V., Bhamidipati, A., Punna, T. et al. (2005) Exploration of the function and organization of the yeast early secretory pathway through an epistatic miniarray profile. Cell 123, 507–519 10.1016/j.cell.2005.08.031 16269340

[19] Costanzo, M., Baryshnikova, A., Bellay, J., Kim, Y., Spear, E.D., Sevier, C.S. et al. (2010) The genetic landscape of a cell. Science 327, 425–431 10.1126/science.1180823 20093466 PMC5600254

[20] Roguev, A., Ryan, C.J., Xu, J., Colson, I., Hartsuiker, E. and Krogan, N. (2016b) Genetic interaction score (S-Score) calculation, clustering, and visualization of genetic interaction profiles for yeast. Cold Spring Harb. Protoc. 10.1101/pdb.prot091983 28733406

[21] Collins, S.R., Schuldiner, M., Krogan, N.J. and Weissman, J.S. (2006) A strategy for extracting and analyzing large-scale quantitative epistatic interaction data. Genome Biol. 7, R63 10.1186/gb-2006-7-7-r63 16859555 PMC1779568

[22] Hartman, J.L., Garvik, B. and Hartwell, L. (2001) Principles for the buffering of genetic variation. Science 291, 1001–1004 10.1126/science.1056072 11232561

[23] Piotrowski, J.S., Li, S.C., Deshpande, R., Simpkins, S.W., Nelson, J., Yashiroda, Y. et al. (2017) Functional annotation of chemical libraries across diverse biological processes. Nat. Chem. Biol. 13, 982–993 10.1038/nchembio.2436 28759014 PMC6056180

[24] Parsons, A.B., Brost, R.L., Ding, H., Li, Z., Zhang, C., Sheikh, B. et al. (2004) Integration of chemical-genetic and genetic interaction data links bioactive compounds to cellular target pathways. Nat. Biotechnol. 22, 62–69 10.1038/nbt919 14661025

[25] Usaj, M., Tan, Y., Wang, W., VanderSluis, B., Zou, A., Myers, C.L. et al. (2017) Thecellmap.org: a web-accessible database for visualizing and mining the global yeast genetic interaction network. G3 (Bethesda) 7, 1539–1549 10.1534/g3.117.040220 28325812 PMC5427489

[26] van Wageningen, S., Kemmeren, P., Lijnzaad, P., Margaritis, T., Benschop, J.J., de Castro, I.J. et al. (2010) Functional overlap and regulatory links shape genetic interactions between signaling pathways. Cell 143, 991–1004 10.1016/j.cell.2010.11.021 21145464 PMC3073509

[27] Sameith, K., Amini, S., Groot Koerkamp, M.J., van Leenen, D., Brok, M., Brabers, N. et al. (2015) A high-resolution gene expression atlas of epistasis between gene-specific transcription factors exposes potential mechanisms for genetic interactions. BMC Biol. 13, 112 10.1186/s12915-015-0222-5 26700642 PMC4690272

[28] Pan, X., Yuan, D.S., Ooi, S.L., Wang, X., Sookhai-Mahadeo, S., Meluh, P. et al. (2007) dSLAM analysis of genome-wide genetic interactions in *Saccharomyces cerevisiae*. Methods 41, 206–221 10.1016/j.ymeth.2006.07.033 17189863 PMC2491713

[29] Decourty, L., Saveanu, C., Zemam, K., Hantraye, F., Frachon, E., Rousselle, J.C. et al. (2008) Linking functionally related genes by sensitive and quantitative characterization of genetic interaction profiles. Proc. Natl Acad. Sci. U.S.A. 105, 5821–5826 10.1073/pnas.0710533105 18408161 PMC2311358

[30] Malabat, C. and Saveanu, C. (2016) Identification of links between cellular pathways by genetic interaction mapping (GIM). Methods Mol. Biol. 1361, 325–343 10.1007/978-1-4939-3079-1_18 26483030

[31] Diaz-Mejia, J.J., Celaj, A., Mellor, J.C., Cote, A., Balint, A., Ho, B. et al. (2018) Mapping DNA damage-dependent genetic interactions in yeast via party mating and barcode fusion genetics. Mol. Syst. Biol. 14, e7985 10.15252/msb.20177985 29807908 PMC5974512

[32] Jaffe, M., Sherlock, G. and Levy, S.F. (2017) Iseq: a new double-barcode method for detecting dynamic genetic interactions in yeast. G3 (Bethesda) 7, 143–153 10.1534/g3.116.034207 27821633 PMC5217104

[33] Sun, S., Baryshnikova, A., Brandt, N. and Gresham, D. (2020) Genetic interaction profiles of regulatory kinases differ between environmental conditions and cellular states. Mol. Syst. Biol. 16, e9167 10.15252/msb.20199167 32449603 PMC7247079

[34] Hanna, R.E. and Doench, J.G. (2020) Design and analysis of CRISPR-Cas experiments. Nat. Biotechnol. 38, 813–823 10.1038/s41587-020-0490-7 32284587

[35] Jaffe, M., Dziulko, A., Smith, J.D., St Onge, R.P., Levy, S.F. and Sherlock, G. (2019) Improved discovery of genetic interactions using CRISPRiSeq across multiple environments. Genome Res. 29, 668–681 10.1101/gr.246603.118 30782640 PMC6442382

[36] Kuzmin, E., VanderSluis, B., Nguyen Ba, A.N., Wang, W., Koch, E.N., Usaj, M. et al. (2020) Exploring whole-genome duplicate gene retention with complex genetic interaction analysis. Science 368, eaaz5667 10.1126/science.aaz5667 32586993 PMC7539174

[37] Kuzmin, E., VanderSluis, B., Wang, W., Tan, G., Deshpande, R., Chen, Y. et al. (2018) Systematic analysis of complex genetic interactions. Science 360, eaao1729 10.1126/science.aao1729 29674565 PMC6215713

[38] Haber, J.E., Braberg, H., Wu, Q., Alexander, R., Haase, J., Ryan, C. et al. (2013) Systematic triple-mutant analysis uncovers functional connectivity between pathways involved in chromosome regulation. Cell Rep. 3, 2168–2178 10.1016/j.celrep.2013.05.007 23746449 PMC3718395

[39] Sanders, E., Nguyen, P.A., Rogers, C.M. and Bochman, M.L. (2020) Comprehensive synthetic genetic array analysis of alleles that interact with mutation of the *Saccharomyces cerevisiae* RecQ helicases Hrq1 and Sgs1. G3 (Bethesda) 10, 4359–4368 10.1534/g3.120.401709 33115720 PMC7718751

[40] Domnauer, M., Zheng, F., Li, L., Zhang, Y., Chang, C.E., Unruh, J.R. et al. (2021) Proteome plasticity in response to persistent environmental change. Mol. Cell 81, 3294–309.e12 10.1016/j.molcel.2021.06.028 34293321 PMC8475771

[41] Costanzo, M., Hou, J., Messier, V., Nelson, J., Rahman, M., VanderSluis, B. et al. (2021) Environmental robustness of the global yeast genetic interaction network. Science 372, eabf8424 10.1126/science.abf8424 33958448 PMC9132594

[42] Celaj, A., Gebbia, M., Musa, L., Cote, A.G., Snider, J., Wong, V. et al. (2020) Highly combinatorial genetic interaction analysis reveals a multi-drug transporter influence network. Cell Syst. 10, 25–38.e10 10.1016/j.cels.2019.09.009 31668799 PMC6989212

[43] Ohya, Y., Sese, J., Yukawa, M., Sano, F., Nakatani, Y., Saito, T.L. et al. (2005) High-dimensional and large-scale phenotyping of yeast mutants. Proc. Natl Acad. Sci. U.S.A. 102, 19015–19020 10.1073/pnas.0509436102 16365294 PMC1316885

[44] Negishi, T., Nogami, S. and Ohya, Y. (2009) Multidimensional quantification of subcellular morphology of *Saccharomyces cerevisiae* using CalMorph, the high-throughput image-processing program. J. Biotechnol. 141, 109–117 10.1016/j.jbiotec.2009.03.014 19433213

[45] Grys, B.T., Lo, D.S., Sahin, N., Kraus, O.Z., Morris, Q., Boone, C. et al. (2017) Machine learning and computer vision approaches for phenotypic profiling. J. Cell Biol. 216, 65–71 10.1083/jcb.201610026 27940887 PMC5223612

[46] Mattiazzi Usaj, M., Lo, D.S., Grys, B.T. and Andrews, B.J. (2021) High-throughput imaging of arrays of fluorescently tagged yeast mutant strains. Methods Mol. Biol. 2304, 221–242 10.1007/978-1-0716-1402-0_12 34028720 PMC9107603

[47] Vizeacoumar, F.J., van Dyk, N., Vizeacoumar, F., Cheung, V., Li, J., Sydorskyy, Y. et al. (2010) Integrating high-throughput genetic interaction mapping and high-content screening to explore yeast spindle morphogenesis. J. Cell Biol. 188, 69–81 10.1083/jcb.200909013 20065090 PMC2812844

[48] Witkin, K.L., Chong, Y., Shao, S., Webster, M.T., Lahiri, S., Walters, A.D. et al. (2012) The budding yeast nuclear envelope adjacent to the nucleolus serves as a membrane sink during mitotic delay. Curr. Biol. 22, 1128–1133 10.1016/j.cub.2012.04.022 22658600 PMC3381997

[49] Walters, A.D., May, C.K., Dauster, E.S., Cinquin, B.P., Smith, E.A., Robellet, X. et al. (2014) The yeast polo kinase Cdc5 regulates the shape of the mitotic nucleus. Curr. Biol. 24, 2861–2867 10.1016/j.cub.2014.10.029 25454593 PMC4255140

[50] Neumuller, R.A., Gross, T., Samsonova, A.A., Vinayagam, A., Buckner, M., Founk, K. et al. (2013) Conserved regulators of nucleolar size revealed by global phenotypic analyses. Sci. Signal. 6, ra70 10.1126/scisignal.2004145 23962978 PMC3964804

[51] Dalton, L.E., Bean, B.D.M., Davey, M. and Conibear, E. (2017) Quantitative high-content imaging identifies novel regulators of Neo1 trafficking at endosomes. Mol. Biol. Cell 28, 1539–1550 10.1091/mbc.e16-11-0772 28404745 PMC5449152

[52] Styles, E.B., Founk, K.J., Zamparo, L.A., Sing, T.L., Altintas, D., Ribeyre, C. et al. (2016) Exploring quantitative yeast phenomics with single-cell analysis of DNA damage foci. Cell Syst. 3, 264–77.e10 10.1016/j.cels.2016.08.008 27617677 PMC5689480

[53] Mattiazzi Usaj, M., Sahin, N., Friesen, H., Pons, C., Usaj, M., Masinas, M.P.D. et al. (2020) Systematic genetics and single-cell imaging reveal widespread morphological pleiotropy and cell-to-cell variability. Mol. Syst. Biol. 16, e9243 10.15252/msb.20199243 32064787 PMC7025093

[54] Burston, H.E., Maldonado-Baez, L., Davey, M., Montpetit, B., Schluter, C., Wendland, B. et al. (2009) Regulators of yeast endocytosis identified by systematic quantitative analysis. J. Cell Biol. 185, 1097–1110 10.1083/jcb.200811116 19506040 PMC2711619

[55] Farrell, K.B., Grossman, C. and Di Pietro, S.M. (2015) New regulators of clathrin-mediated endocytosis identified in *Saccharomyces cerevisiae* by systematic quantitative fluorescence microscopy. Genetics 201, 1061–1070 10.1534/genetics.115.180729 26362318 PMC4649635

[56] Jo, M.C., Liu, W., Gu, L., Dang, W. and Qin, L. (2015) High-throughput analysis of yeast replicative aging using a microfluidic system. Proc. Natl Acad. Sci. U.S.A. 112, 9364–9369 10.1073/pnas.1510328112 26170317 PMC4522780

[57] Lee, S.S., Avalos Vizcarra, I., Huberts, D.H., Lee, L.P. and Heinemann, M. (2012) Whole lifespan microscopic observation of budding yeast aging through a microfluidic dissection platform. Proc. Natl Acad. Sci. U.S.A. 109, 4916–4920 10.1073/pnas.1113505109 22421136 PMC3324001

[58] Huh, W.K., Falvo, J.V., Gerke, L.C., Carroll, A.S., Howson, R.W., Weissman, J.S. et al. (2003) Global analysis of protein localization in budding yeast. Nature 425, 686–691 10.1038/nature02026 14562095

[59] Ho, B., Baryshnikova, A. and Brown, G.W. (2018) Unification of protein abundance datasets yields a quantitative *Saccharomyces cerevisiae* proteome. Cell Syst. 6, 192–205.e3 10.1016/j.cels.2017.12.004 29361465

[60] Chong, Y.T., Koh, J.L., Friesen, H., Duffy, S.K., Cox, M.J., Moses, A. et al. (2015) Yeast proteome dynamics from single cell imaging and automated analysis. Cell 161, 1413–1424 10.1016/j.cell.2015.04.051 26046442

[61] Breker, M., Gymrek, M. and Schuldiner, M. (2013) A novel single-cell screening platform reveals proteome plasticity during yeast stress responses. J. Cell Biol. 200, 839–850 10.1083/jcb.201301120 23509072 PMC3601363

[62] Weill, U., Krieger, G., Avihou, Z., Milo, R., Schuldiner, M. and Davidi, D. (2019) Assessment of GFP Tag position on protein localization and growth fitness in yeast. J. Mol. Biol. 431, 636–641 10.1016/j.jmb.2018.12.004 30550779 PMC7611381

[63] Weill, U., Yofe, I., Sass, E., Stynen, B., Davidi, D., Natarajan, J. et al. (2018) Genome-wide SWAp-Tag yeast libraries for proteome exploration. Nat. Methods 15, 617–622 10.1038/s41592-018-0044-9 29988094 PMC6076999

[64] Yofe, I., Weill, U., Meurer, M., Chuartzman, S., Zalckvar, E., Goldman, O. et al. (2016) One library to make them all: streamlining the creation of yeast libraries via a SWAp-Tag strategy. Nat. Methods 13, 371–378 10.1038/nmeth.3795 26928762 PMC4869835

[65] Dubreuil, B., Sass, E., Nadav, Y., Heidenreich, M., Georgeson, J.M., Weill, U. et al. (2019) YeastRGB: comparing the abundance and localization of yeast proteins across cells and libraries. Nucleic Acids Res. 47, D1245–D12D9 10.1093/nar/gky941 30357397 PMC6324022

[66] Meurer, M., Duan, Y., Sass, E., Kats, I., Herbst, K., Buchmuller, B.C. et al. (2018) Genome-wide C-SWAT library for high-throughput yeast genome tagging. Nat. Methods 15, 598–600 10.1038/s41592-018-0045-8 29988096

[67] Khmelinskii, A. and Knop, M. (2014) Analysis of protein dynamics with tandem fluorescent protein timers. Methods Mol. Biol. 1174, 195–210 10.1007/978-1-4939-0944-5_13 24947383

[68] Kraus, O.Z., Grys, B.T., Ba, J., Chong, Y., Frey, B.J., Boone, C. et al. (2017) Automated analysis of high-content microscopy data with deep learning. Mol. Syst. Biol. 13, 924 10.15252/msb.20177551 28420678 PMC5408780

[69] Parnamaa, T. and Parts, L. (2017) Accurate classification of protein subcellular localization from high-throughput microscopy images using deep learning. G3 (Bethesda) 7, 1385–1392 10.1534/g3.116.033654 28391243 PMC5427497

[70] Mattiazzi Usaj, M., Yeung, C.H.L., Friesen, H., Boone, C. and Andrews, B.J. (2021) Single-cell image analysis to explore cell-to-cell heterogeneity in isogenic populations. Cell Syst. 12, 608–621 10.1016/j.cels.2021.05.010 34139168 PMC9112900

